# Protection of pipeline below pavement subjected to traffic induced dynamic response

**DOI:** 10.1038/s41598-023-31615-7

**Published:** 2023-03-27

**Authors:** Chaidul Haque Chaudhuri, Deepankar Choudhury

**Affiliations:** Department of Civil Engineering, Indian Institute of Technology Bombay, Powai, Mumbai 400076 India

**Keywords:** Civil engineering, Applied mathematics, Software

## Abstract

Failure of pipelines below road pavement results to the disruption of both the traffic movement and the consumers of the pipelines. Intermediate safeguard layer can be used to protect the pipeline from heavy traffic loads. The present study proposed analytical solutions to obtain the dynamic response of buried pipe below road pavement with and without considering safeguard based on the concept of triple and double beam system respectively. Pavement layer, safeguard and the pipeline are considered as Euler Bernoulli’s beam. Advanced soil model is used (viscoelastic foundation with shear interaction between springs) to model the surrounding soil. Self-weight of soil is also considered in the present study. The obtained governing coupled differential equations are solved adopting finite sine Fourier transform, Laplace transform and their inverse transformation. The proposed formulation is initially verified with the past numerical and analytical studies and then validated with the three-dimensional finite element based numerical analysis. From parametric study it is perceived that the stability of the pipe can be significantly increased by providing intermediate barrier. Further, pipe deformation is increases with increasing traffic loads. At very high-speed range (> 60 m s^−1^), pipe deformation is significantly rises with increasing traffic speed. The present study can be useful in preliminary design stage before performing rigorous and expensive numerical or experimental study.

## Introduction

Pipelines act as veins across the country and are the main means of transportation for a variety of things including water, oil, natural gas, telecommunication and electricity lines. Pipelines are generally buried below the ground surface due to the scarcity of unused land, to maintain smooth operation of modern urbanization and to protect from damage due to vandalism. In urban areas pipelines are often placed below the road pavement. Deformation of pipelines below the road pavement due to moving traffic load cause the inconvenience of the traffic movement. Further, if pipeline fails it may lead to the disruption of whole pipe network, discomfort of the consumers, source of firing and leads to a disaster depending on the substance carrying by the pipelines. For viz. failure of water pipeline below the pavement may results small leaks without effecting the pavement, water leakage through the pavement, uplifts the pavement, or cavity generation below the pavement^1,2^. Hence, it is essential to ensure the safety of the underground pipelines below the road pavement. The pipeline should resist both the overburden soil pressure and live traffic loads. Alzabeebee et al.^3,4^ carried out 3D numerical analysis to investigate the response of polyvinyl chloride (PVC) and concrete pipe under UK standard traffic loads. The combined effect of traffic load and ground water fluctuations on underground concrete pipes has been investigated by Li et al.^5^ through 3D finite element based numerical analysis. It was observed that the pipe stress and vertical deflection are directly proportional to the permeability co-efficient and void ratio. Alzabeebee et al.^6^ performed a comparative numerical analysis to study the impact of static and moving traffic loads on buried pipes. From numerical analysis, it was observed that the effect of soil plasticity on pipe response is negligible for the particular adopted condition and static traffic loads provides higher pipe deformation compared to moving traffic loads. Further, Xu et al.^7^ examined the longitudinal response of a 1.4 m diameter jointed (gasket, bell and spigot) reinforced concrete pipeline under traffic loads. The study was carried out numerically using finite difference based program FLAC-3D. It was noticed that the pipe response significantly changes with soil stiffness. However, the impact of gasket stiffness on longitudinal pipe response was minor. The influence of moving traffic load on the response of buried pipe in cohesionless soil was studied through both 3D numerical and centrifuge modeling by Saboya et al.^8^. Rakitin and Xu^9^ performed centrifuge tests on large diameter (1.4 m) buried pipelines under heavy traffic loads (up to 850 kN). Maximum unfavorable condition was achieved when the heaviest axle was just above the pipe crown. With increasing soil cover depth initial bending moment of the pipe was increased due to soil weight but moment due to traffic load was significantly reduced. The behaviour of buried culvert subjected to different loading condition including traffic loading is also assessed by performing field test and full-scale laboratory test^10,11^. However, underground utilities (pipe, culvert) can be protected from external live loads such as traffic loads or loads generated from permanent ground deformation by providing suitable barrier system. For instance, EPS geofoam can be used as a barrier to protect underground pipes or culverts from additional stresses induced from external dead or live loads^12^. Concrete cap or protective reinforced slab can also be used to protect the pipeline below highway from surface loads or dig-ups^13^. Moreover, experimental and numerical studies are conducted on geocell reinforced soil to protect the underground pipelines from traffic loads^14–17^. Another important aspect of underground projects is the rockburst incident. Researchers are proposed various prediction model to predict the rockburst hazard^18–20^. Robert et al.^21^ conducted both field and numerical study to obtain the response of underground water pipes subjected to traffic loads.

Apart from full scale experimental and three-dimensional numerical analyses, another alternative way of solving complex soil-structure interaction related problem in a simplified way is the analytical approach which is based on beam spring concept. For instance, Kausel et al.^22^ investigated the critical speed of high-speed rail considering the theory of beam on elastic foundation. The limitation of the study is that it did not incorporate the system damping and any shear interaction between elastic springs. Yin^23^ performed an analytical study for reinforced beam on single parameter elastic foundation subjected to a point load. Chaudhuri and Choudhury^24–26^ proposed different simplified theoretical solutions for buried pipe subjected to ground deformation resulting from seismic landslide, horizontal transverse ground deformation, and static pipe bursting underneath respectively. The prior studies were conducted considering pipe as Euler Bernoulli’s or Timoshenko beam and soil as single parameter Winkler or 2-parameter Pasternak foundation. Adopting the similar concept of beam-spring model Wu et al.^27^ and Chaudhuri and Choudhury^28^ examined the dynamic behaviour of buried pipe subjected to surface and subsurface blast loads respectively. Further, Zhang et al.^29,30^ investigated the response of pavement structure on geocell reinforced embankment under vehicle loads (concentrated loads and moving load respectively) using double beam model. As per the author’s knowledge closed-form analytical study on pipe below road pavement with and without intermediate safeguard is not available in the literature. In this regard, the present study proposed closed form solutions for buried pipe with and without protective layer below the pavement structure subjected to moving traffic load by idealizing the problem as triple and double beam system respectively. Soil is idealized as advanced soil model, i.e., viscoelastic foundation with shear interaction between distinct springs. The self-weight of the soil above the pipe and the safeguard layer is also considered. After conducting verification studies with the past numerical and analytical works and a validation study with three-dimensional finite element based numerical analysis, a parametric study is carried out to understand the advantage of protective layer and the impact of traffic load and traffic velocity on pipe response for both barrier and without barrier system.

The main contribution of the present study is the proposed closed-form solutions to obtain the response of pipe under traffic load with and without considering intermediate safeguard layer. In literature, no such analytical solutions are available. In industrial applications or in the preliminary design stage of buried pipe below road pavement, proposed solutions can be used to get quick and approximate results with moderate accuracy. Simply putting the input values and following the flowchart as depicted in Fig. 2, one can easily obtain the pipe response quickly. The analytical solution provides advantages from an economic standpoint since it requires fewer input parameters, is easier to understand, and takes less time. Therefore, in the basic design stage, such analytical solutions can be used before conducting a thorough numerical or experimental study.

## Problem definition

**Figure 1:**
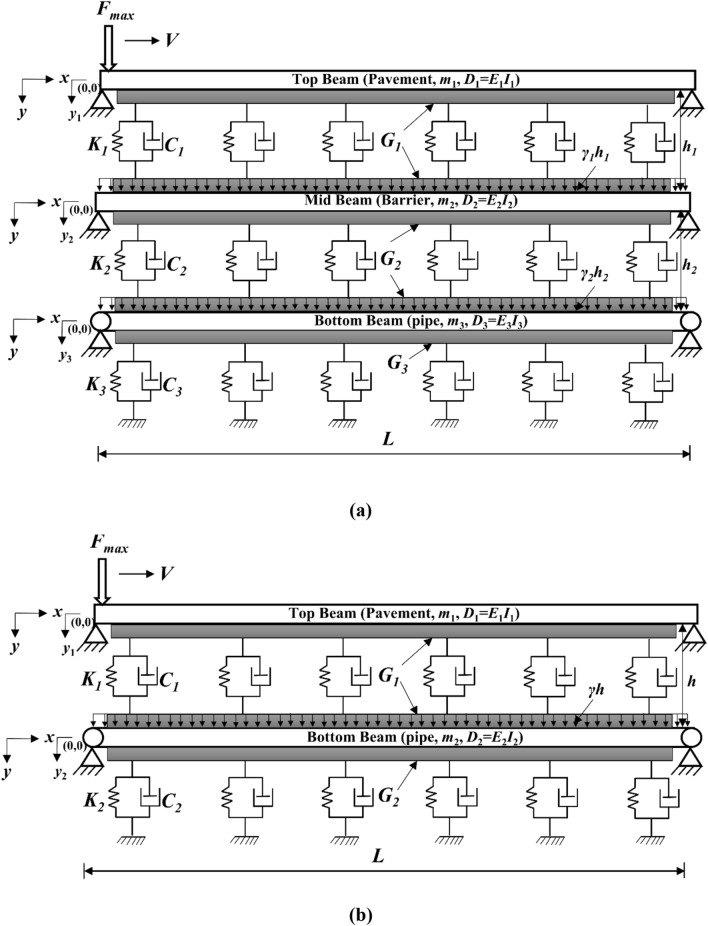
Analytical model of pipe below the pavement structure (**a**) with safeguard and (**b**) without safeguard.

Figure 1a,b depicts the idealization of buried pipe with and without protection layer below the road pavement respectively. The pavement layer, barrier and the pipe are considered as Euler Bernoulli’s beam and the soil is simulated using viscoelastic foundation with shear interaction between individual springs. The self-weight of soil is also acknowledged in the analysis. The top beam (pavement structure) is subjected to moving concentrated force to simulate the moving traffic load. The end boundary conditions of each beam are considered as simply supported and the length of the beam (*L*) is taken as sufficiently long to avoid the influence of boundary conditions on peak pipe responses. The present study proposed a generalized formulation considering different layers of soil in between the beams. In Fig. 1a the stiffness of soil springs, damping co-efficient and shear of the interaction layer of top, mid and bottom layers are represented as *K*_1_,* K*_2_, *K*_3_, *C*_1_, *C*_2_, *C*_3_, and *G*_1_, *G*_2_, *G*_3_ respectively. *m*_1_, *m*_2_, *m*_3_ and *D*_1_, *D*_2_,* D*_3_ are the mass per unit length and flexural rigidity of the top, mid and bottom beam respectively. γ_1_, γ_2_ and* h*_1_, *h*_2_ are the unit weight and thickness of the top and mid soil layer respectively. Similarly, in Fig. 1b the stiffness of soil springs, damping co-efficient and shear of the interaction layer of top and bottom layers are represented as *K*_1_, *K*_2_, *C*_1_, *C*_2_ and *G*_1_, *G*_2_ respectively. *m*_1_, *m*_2_ and *D*_1_, *D*_2_ are the mass per unit length and flexural rigidity of the top, and bottom beam respectively. γ and *h* are the unit weight and thickness of the top soil layer respectively. For simplicity, the outputs of the present study are obtained considering identical soil stiffness. Following equations are used for calculating soil stiffness, shear parameter and damping coefficients^30–32^.

1 $$K_{1} = K_{2} = K_{3} = \frac{{E_{s} B\gamma \left( {1 - \nu_{s} } \right)}}{{2\left( {1 + \nu_{s} } \right)\left( {1 - 2\nu_{s} } \right)}} \,$$

2 $$G_{1} = G_{2} = G_{3} = \frac{{2E_{s} B}}{{8\gamma \left( {1 + \nu_{s} } \right)}}$$

3 $$C_{i} = 2\xi_{i} \sqrt {K_{i} m_{i} } \, i = 1,2,3{\text{ (triple beam) and }}i = 1,2{\text{ (double beam)}}$$

where *E*_s_ and *B* are the Young’s modulus of soil and width of the beam respectively, γ is the rate at which the vertical displacement in the ground diminishes with depth, ν_s_ and ξ are the Poisson’s ratio of soil and damping ratio respectively.

## Coupled differential equations and analytical solutions

The current study proposed analytical solutions for the buried pipe with and without safeguard below the road pavement. The coupled differential equations and corresponding solutions for both the prior mentioned cases are stated in the subsequent sections.

### Pipe with safeguard

The governing coupled differential equations of top (pavement), mid (safeguard) and bottom (pipe) beam as shown in Fig. 1a can be expressed as

4 $$D_{1} \frac{{\partial^{4} y_{1} }}{{\partial x^{4} }} - G_{1} \left( {\frac{{\partial^{2} y_{1} }}{{\partial x^{2} }} - \frac{{\partial^{2} y_{2} }}{{\partial x^{2} }}} \right) + m_{1} \frac{{\partial^{2} y_{1} }}{{\partial t^{2} }} + C_{1} \left( {\frac{{\partial y_{1} }}{\partial t} - \frac{{\partial y_{2} }}{\partial t}} \right) + K_{1} (y_{1} - y_{2} ) = F_{\max } \delta (x - Vt)$$

5 $$\begin{gathered} D_{2} \frac{{\partial^{4} y_{2} }}{{\partial x^{4} }} + G_{1} \left( {\frac{{\partial^{2} y_{1} }}{{\partial x^{2} }} - \frac{{\partial^{2} y_{2} }}{{\partial x^{2} }}} \right) + G_{2} \left( {\frac{{\partial^{2} y_{3} }}{{\partial x^{2} }} - \frac{{\partial^{2} y_{2} }}{{\partial x^{2} }}} \right) + m_{2} \frac{{\partial^{2} y_{2} }}{{\partial t^{2} }} - C_{1} \left( {\frac{{\partial y_{1} }}{\partial t} - \frac{{\partial y_{2} }}{\partial t}} \right) \hfill \\ + C_{2} \left( {\frac{{\partial y_{2} }}{\partial t} - \frac{{\partial y_{3} }}{\partial t}} \right) - K_{1} (y_{1} - y_{2} ) + K_{2} (y_{2} - y_{3} ) = \gamma_{1} h_{1} B \hfill \\ \end{gathered}$$

6 $$\begin{gathered} D_{3} \frac{{\partial^{4} y_{3} }}{{\partial x^{4} }} + G_{2} \left( {\frac{{\partial^{2} y_{2} }}{{\partial x^{2} }} - \frac{{\partial^{2} y_{3} }}{{\partial x^{2} }}} \right) - G_{3} \frac{{\partial^{2} y_{3} }}{{\partial x^{2} }} + m_{3} \frac{{\partial^{2} y_{3} }}{{\partial t^{2} }} - C_{2} \left( {\frac{{\partial y_{2} }}{\partial t} - \frac{{\partial y_{3} }}{\partial t}} \right) \hfill \\ + C_{3} \frac{{\partial y_{3} }}{\partial t} - K_{2} (y_{2} - y_{3} ) + K_{3} y_{3} = \gamma_{2} h_{2} \frac{\pi D}{2} \hfill \\ \end{gathered}$$

where *F*_max_ is the maximum traffic load and *V* is the traffic velocity.

First, finite sine Fourier transform is applied to solve the coupled differential equations. The finite sine Fourier transform for spatial co-ordinate *x* $(0\le x\le L)$ and its inverse can be defined as follows

7 $$F[y(x,t)] = Y(\xi_{n} ,t) = \int\limits_{0}^{L} {y(x,t)\sin (\xi_{n} x)dx}$$

8 $$F^{ - 1} [Y(\xi_{n} ,t)] = y(x,t) = \frac{2}{L}\sum\limits_{n = 1}^{\infty } {Y(\xi_{n} ,t)} \sin (\xi_{n} x)$$

where $$\xi_{n} = \frac{n\pi }{L}$$ and *n* = 1, 2, 3, ….

For simply supported beams as shown in Fig. 1a, the boundary conditions are,

9 $$y_{k} \left( {0,t} \right) = y_{k} \left( {L,t} \right) = 0{\text{ where }}k = 1,2,3$$

10 $$E_{k} I_{k} \frac{{\partial^{2} y_{k} \left( {0,t} \right)}}{{\partial x^{2} }} = E_{k} I_{k} \frac{{\partial^{2} y_{k} \left( {L,t} \right)}}{{\partial x^{2} }} = 0{\text{ where }}k = 1,2,3$$

The following equations can be obtained after performing finite Sine-Fourier transform on both sides of Eqs. (4)–(6)

11 $$\begin{gathered} D_{1} \xi_{n}^{4} Y_{1} (\xi_{n} ,t) + G_{1} \xi_{n}^{2} \left\{ {Y_{1} (\xi_{n} ,t) - Y_{2} (\xi_{n} ,t)} \right\} + m_{1} \frac{{\partial^{2} }}{{\partial t^{2} }}Y_{1} (\xi_{n} ,t) + C_{1} \frac{\partial }{\partial t}\left\{ {Y_{1} (\xi_{n} ,t) - Y_{2} (\xi_{n} ,t)} \right\} \hfill \\ + K_{1} \left\{ {Y_{1} (\xi_{n} ,t) - Y_{2} (\xi_{n} ,t)} \right\} = F_{\max } \sin (\xi_{n} Vt) \hfill \\ \end{gathered}$$

12 $$\begin{gathered} D_{2} \xi_{n}^{4} Y_{2} (\xi_{n} ,t) - G_{1} \xi_{n}^{2} \left\{ {Y_{1} (\xi_{n} ,t) - Y_{2} (\xi_{n} ,t)} \right\} - G_{2} \xi_{n}^{2} \left\{ {Y_{3} (\xi_{n} ,t) - Y_{2} (\xi_{n} ,t)} \right\} + \hfill \\ m_{2} \frac{{\partial^{2} }}{{\partial t^{2} }}Y_{2} (\xi_{n} ,t) - C_{1} \frac{\partial }{\partial t}\left\{ {Y_{1} (\xi_{n} ,t) - Y_{2} (\xi_{n} ,t)} \right\} + C_{2} \frac{\partial }{\partial t}\left\{ {Y_{2} (\xi_{n} ,t) - Y_{3} (\xi_{n} ,t)} \right\} - \hfill \\ K_{1} \left\{ {Y_{1} (\xi_{n} ,t) - Y_{2} (\xi_{n} ,t)} \right\} + K_{2} \left\{ {Y_{2} (\xi_{n} ,t) - Y_{3} (\xi_{n} ,t)} \right\} = \gamma_{1} h_{1} B\frac{1}{{\xi_{n} }}\left\{ {1 - \cos (\xi_{n} L)} \right\} \hfill \\ \end{gathered}$$

13 $$\begin{gathered} D_{3} \xi_{n}^{4} Y_{3} (\xi_{n} ,t) - G_{2} \xi_{n}^{2} \left\{ {Y_{2} (\xi_{n} ,t) - Y_{3} (\xi_{n} ,t)} \right\} + G_{3} \xi_{n}^{2} Y_{3} (\xi_{n} ,t) + \hfill \\ m_{3} \frac{{\partial^{2} }}{{\partial t^{2} }}Y_{3} (\xi_{n} ,t) - C_{2} \frac{\partial }{\partial t}\left\{ {Y_{2} (\xi_{n} ,t) - Y_{3} (\xi_{n} ,t)} \right\} + C_{3} \frac{\partial }{\partial t}Y_{3} (\xi_{n} ,t) - \hfill \\ K_{2} \left\{ {Y_{2} (\xi_{n} ,t) - Y_{3} (\xi_{n} ,t)} \right\} + K_{3} Y_{3} (\xi_{n} ,t) = \gamma_{2} h_{2} \frac{\pi D}{{2\xi_{n} }}\left\{ {1 - \cos (\xi_{n} L)} \right\} \hfill \\ \end{gathered}$$

Further, Laplace transformation of first and second derivative of displacement with respect to time, *t* can be expressed as

14 $${\text{L}}\left[ {\frac{{{\text{d}}y(t)}}{{{\text{d}}t}}} \right] = s{\text{L}}\left[ {y(t)} \right] - y(0)$$

15 $${\text{L}}\left[ {\frac{{{\text{d}}^{2} y(t)}}{{{\text{d}}t^{2} }}} \right] = s^{2} {\text{L}}\left[ {y(t)} \right] - sy(0) - \frac{{{\text{d}}y}}{{{\text{d}}t}}_{t = 0}$$

where, *s* is the transformed variable of time (*t*). At initial condition (i.e., *t* = 0) both displacement and velocity will be zero. Taking Laplace transformation with respect to *t* on both sides of Eqs. (11)–(13), the succeeding equations can be obtained

16 $$(m_{1} s^{2} + C_{1} s + K_{1} + G_{1} \xi_{n}^{2} + D_{1} \xi_{n}^{4} )Y_{1}^{ * } (\xi_{n} ,s) - (C_{1} s + K_{1} + G_{1} \xi_{n}^{2} )Y_{2}^{ * } (\xi_{n} ,s) = R_{1} (\xi_{n} ,s)$$

17 $$\begin{gathered} - (C_{1} s + K_{1} + G_{1} \xi_{n}^{2} )Y_{1}^{ * } (\xi_{n} ,s) + (m_{2} s^{2} + C_{1} s + C_{2} s + K_{1} + K_{2} + G_{1} \xi_{n}^{2} + \hfill \\ G_{2} \xi_{n}^{2} + D_{2} \xi_{n}^{4} )Y_{2}^{ * } (\xi_{n} ,s) - (C_{2} s + K_{2} + G_{2} \xi_{n}^{2} )Y_{3}^{ * } (\xi_{n} ,s) = R_{2} (\xi_{n} ,s) \hfill \\ \end{gathered}$$

18 $$\begin{gathered} - (C_{2} s + K_{2} + G_{2} \xi_{n}^{2} )Y_{2}^{ * } (\xi_{n} ,s) + (m_{3} s^{2} + C_{2} s + C_{3} s + K_{2} + K_{3} + G_{2} \xi_{n}^{2} + \hfill \\ G_{3} \xi_{n}^{2} + D_{3} \xi_{n}^{4} )Y_{3}^{ * } (\xi_{n} ,s) = R_{3} (\xi_{n} ,s) \hfill \\ \end{gathered}$$

where, $$R_{1} (\xi_{n} ,s) = L\left[ {F_{\max } \sin (\xi_{n} Vt)} \right]$$; $$R_{2} (\xi_{n} ,s) = L\left[ {\gamma_{1} h_{1} B\frac{1}{{\xi_{n} }}\left\{ {1 - \cos (\xi_{n} L)} \right\}} \right]$$; and $$R_{3} (\xi_{n} ,s) = L\left[ {\gamma_{2} h_{2} \frac{\pi D}{{2\xi_{n} }}\left\{ {1 - \cos (\xi_{n} L)} \right\}} \right]$$.

Equations (16)–(18) can further be written as

19 $$\left[ D \right]_{3 \times 3} \left\{ {\begin{array}{*{20}c} {Y_{1}^{ * } (\xi_{n} ,s)} \\ {Y_{2}^{ * } (\xi_{n} ,s)} \\ {Y_{3}^{ * } (\xi_{n} ,s)} \\ \end{array} } \right\}_{3 \times 1} = \left\{ {\begin{array}{*{20}c} {R_{1} (\xi_{n} ,s)} \\ {R_{2} (\xi_{n} ,s)} \\ {R_{3} (\xi_{n} ,s)} \\ \end{array} } \right\}_{3 \times 1}$$

where

20 $$\left[ D \right]_{3 \times 3} = \left\{ {\begin{array}{*{20}c} {m_{1} s^{2} + C_{1} s + K_{1} + G_{1} \xi_{n}^{2} + D_{1} \xi_{n}^{4} } & { - (C_{1} s + K_{1} + G_{1} \xi_{n}^{2} )} & 0 \\ { - (C_{1} s + K_{1} + G_{1} \xi_{n}^{2} )} & \begin{gathered} m_{2} s^{2} + C_{1} s + C_{2} s + K_{1} + K_{2} + G_{1} \xi_{n}^{2} + \hfill \\ G_{2} \xi_{n}^{2} + D_{2} \xi_{n}^{4} \hfill \\ \end{gathered} & { - (C_{2} s + K_{2} + G_{2} \xi_{n}^{2} )} \\ 0 & { - (C_{2} s + K_{2} + G_{2} \xi_{n}^{2} )} & \begin{gathered} m_{3} s^{2} + C_{2} s + C_{3} s + K_{2} + K_{3} + G_{2} \xi_{n}^{2} + \hfill \\ G_{3} \xi_{n}^{2} + D_{3} \xi_{n}^{4} \hfill \\ \end{gathered} \\ \end{array} } \right\}_{3 \times 3}$$

Now, performing finite Sine-Fourier inverse transformation following expression is obtained

21 $$y_{i} (x,s) = \frac{2}{L}\sum\limits_{n = 1}^{\infty } {Y_{i}^{*} (\xi_{n} ,s)} \sin (\xi_{n} x) \, i = 1,2,3 \,$$

Finally, beam’s deflection response in space and time domain can be obtained by conducting Laplace inverse transformation of Eq. (21)

22 $$y_{i} (x,t) = L^{ - 1} \left[ {y_{i} (x,s)} \right] \, i = 1,2,3 \,$$

### Pipe without safeguard

For the case of pipe without safeguard as shown in Fig. 1b, the governing coupled partial differential equations of top (pavement) and bottom (pipe) beam can be expressed as

23 $$D_{1} \frac{{\partial^{4} y_{1} }}{{\partial x^{4} }} - G_{1} \left( {\frac{{\partial^{2} y_{1} }}{{\partial x^{2} }} - \frac{{\partial^{2} y_{2} }}{{\partial x^{2} }}} \right) + m_{1} \frac{{\partial^{2} y_{1} }}{{\partial t^{2} }} + C_{1} \left( {\frac{{\partial y_{1} }}{\partial t} - \frac{{\partial y_{2} }}{\partial t}} \right) + K_{1} (y_{1} - y_{2} ) = F_{\max } \delta (x - Vt)$$

24 $$\begin{gathered} D_{2} \frac{{\partial^{4} y_{2} }}{{\partial x^{4} }} + G_{1} \left( {\frac{{\partial^{2} y_{1} }}{{\partial x^{2} }} - \frac{{\partial^{2} y_{2} }}{{\partial x^{2} }}} \right) - G_{2} \frac{{\partial^{2} y_{2} }}{{\partial x^{2} }} + m_{2} \frac{{\partial^{2} y_{2} }}{{\partial t^{2} }} - C_{1} \left( {\frac{{\partial y_{1} }}{\partial t} - \frac{{\partial y_{2} }}{\partial t}} \right) \hfill \\ + C_{2} \frac{{\partial y_{2} }}{\partial t} - K_{1} (y_{1} - y_{2} ) + K_{2} y_{2} = \gamma h\frac{\pi D}{2} \hfill \\ \end{gathered}$$

Similar to preceding section, for simply supported beams (Fig. 1b), the available boundary conditions are

25 $$y_{k} \left( {0,t} \right) = y_{k} \left( {L,t} \right) = 0{\text{ where }}k = 1,2$$

26 $$E_{k} I_{k} \frac{{\partial^{2} y_{k} \left( {0,t} \right)}}{{\partial x^{2} }} = E_{k} I_{k} \frac{{\partial^{2} y_{k} \left( {L,t} \right)}}{{\partial x^{2} }} = 0{\text{ where }}k = 1,2$$

Applying finite Sine-Fourier transform on both sides of Eqs. (23) and (24), following equations are obtained

27 $$\begin{gathered} D_{1} \xi_{n}^{4} Y_{1} (\xi_{n} ,t) + G_{1} \xi_{n}^{2} \left\{ {Y_{1} (\xi_{n} ,t) - Y_{2} (\xi_{n} ,t)} \right\} + m_{1} \frac{{\partial^{2} }}{{\partial t^{2} }}Y_{1} (\xi_{n} ,t) + C_{1} \frac{\partial }{\partial t}\left\{ {Y_{1} (\xi_{n} ,t) - Y_{2} (\xi_{n} ,t)} \right\} \hfill \\ + K_{1} \left\{ {Y_{1} (\xi_{n} ,t) - Y_{2} (\xi_{n} ,t)} \right\} = F_{\max } \sin (\xi_{n} Vt) \hfill \\ \end{gathered}$$

28 $$\begin{gathered} D_{2} \xi_{n}^{4} Y_{2} (\xi_{n} ,t) - G_{1} \xi_{n}^{2} \left\{ {Y_{1} (\xi_{n} ,t) - Y_{2} (\xi_{n} ,t)} \right\} + G_{2} \xi_{n}^{2} Y_{2} (\xi_{n} ,t) + \hfill \\ m_{2} \frac{{\partial^{2} }}{{\partial t^{2} }}Y_{2} (\xi_{n} ,t) - C_{1} \frac{\partial }{\partial t}\left\{ {Y_{1} (\xi_{n} ,t) - Y_{2} (\xi_{n} ,t)} \right\} + C_{2} \frac{\partial }{\partial t}Y_{2} (\xi_{n} ,t) - \hfill \\ K_{1} \left\{ {Y_{1} (\xi_{n} ,t) - Y_{2} (\xi_{n} ,t)} \right\} + K_{2} Y_{2} (\xi_{n} ,t) = \gamma h\frac{\pi D}{{2\xi_{n} }}\left\{ {1 - \cos (\xi_{n} L)} \right\} \hfill \\ \end{gathered}$$

Taking Laplace transformation with respect to *t* on both sides of Eqs. (27) and (28),

29 $$(m_{1} s^{2} + C_{1} s + K_{1} + G_{1} \xi_{n}^{2} + D_{1} \xi_{n}^{4} )Y_{1}^{ * } (\xi_{n} ,s) - (C_{1} s + K_{1} + G_{1} \xi_{n}^{2} )Y_{2}^{ * } (\xi_{n} ,s) = R_{1} (\xi_{n} ,s)$$

30 $$\begin{gathered} - (C_{1} s + K_{1} + G_{1} \xi_{n}^{2} )Y_{1}^{ * } (\xi_{n} ,s) + (m_{2} s^{2} + C_{1} s + C_{2} s + K_{1} + K_{2} + G_{1} \xi_{n}^{2} + \hfill \\ G_{2} \xi_{n}^{2} + D_{2} \xi_{n}^{4} )Y_{2}^{ * } (\xi_{n} ,s) = R_{2} (\xi_{n} ,s) \hfill \\ \end{gathered}$$

where, $$R_{1} (\xi_{n} ,s) = L\left[ {F_{\max } \sin (\xi_{n} Vt)} \right]$$; and $$R_{2} (\xi_{n} ,s) = L\left[ {\gamma h\frac{\pi D}{{2\xi_{n} }}\left\{ {1 - \cos (\xi_{n} L)} \right\}} \right]$$;

Equations (29)–(30) can further be reduced as

31 $$\left[ D \right]_{2 \times 2} \left\{ {\begin{array}{*{20}c} {Y_{1}^{ * } (\xi_{n} ,s)} \\ {Y_{2}^{ * } (\xi_{n} ,s)} \\ \end{array} } \right\}_{2 \times 1} = \left\{ {\begin{array}{*{20}c} {R_{1} (\xi_{n} ,s)} \\ {R_{2} (\xi_{n} ,s)} \\ \end{array} } \right\}_{2 \times 1}$$

where

32 $$\left[ D \right]_{2 \times 2} = \left\{ {\begin{array}{*{20}c} {m_{1} s^{2} + C_{1} s + K_{1} + G_{1} \xi_{n}^{2} + D_{1} \xi_{n}^{4} } & { - (C_{1} s + K_{1} + G_{1} \xi_{n}^{2} )} \\ { - (C_{1} s + K_{1} + G_{1} \xi_{n}^{2} )} & \begin{gathered} m_{2} s^{2} + C_{1} s + C_{2} s + K_{1} + K_{2} + G_{1} \xi_{n}^{2} + \hfill \\ G_{2} \xi_{n}^{2} + D_{2} \xi_{n}^{4} \hfill \\ \end{gathered} \\ \end{array} } \right\}_{2 \times 2}$$

Performing finite Sine-Fourier inverse transformation

33 $$y_{i} (x,s) = \frac{2}{L}\sum\limits_{n = 1}^{\infty } {Y_{i}^{*} (\xi_{n} ,s)} \sin (\xi_{n} x) \, i = 1,2$$

Now performing Laplace inverse transformation of Eq. (33), following expression of beam deflection in space–time domain is obtained

34 $$y_{i} (x,t) = L^{ - 1} \left[ {y_{i} (x,s)} \right] \, i = 1,2$$

**Figure 2:**
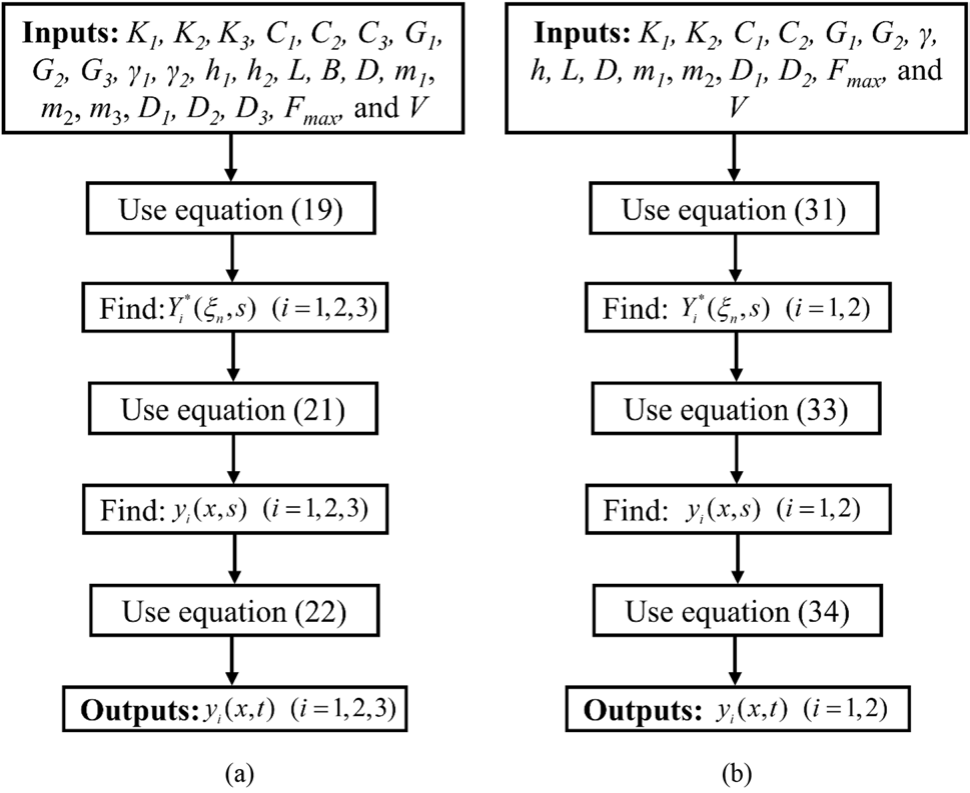
Flowchart of the proposed methodology (**a**) with safeguard and (**b**) without safeguard.

The flowchart of the present methodology for both pipe with and without safeguard below pavement subjected to traffic load is depicted in Fig. 2.

## Verification and validation of proposed analytical study

**Figure 3:**
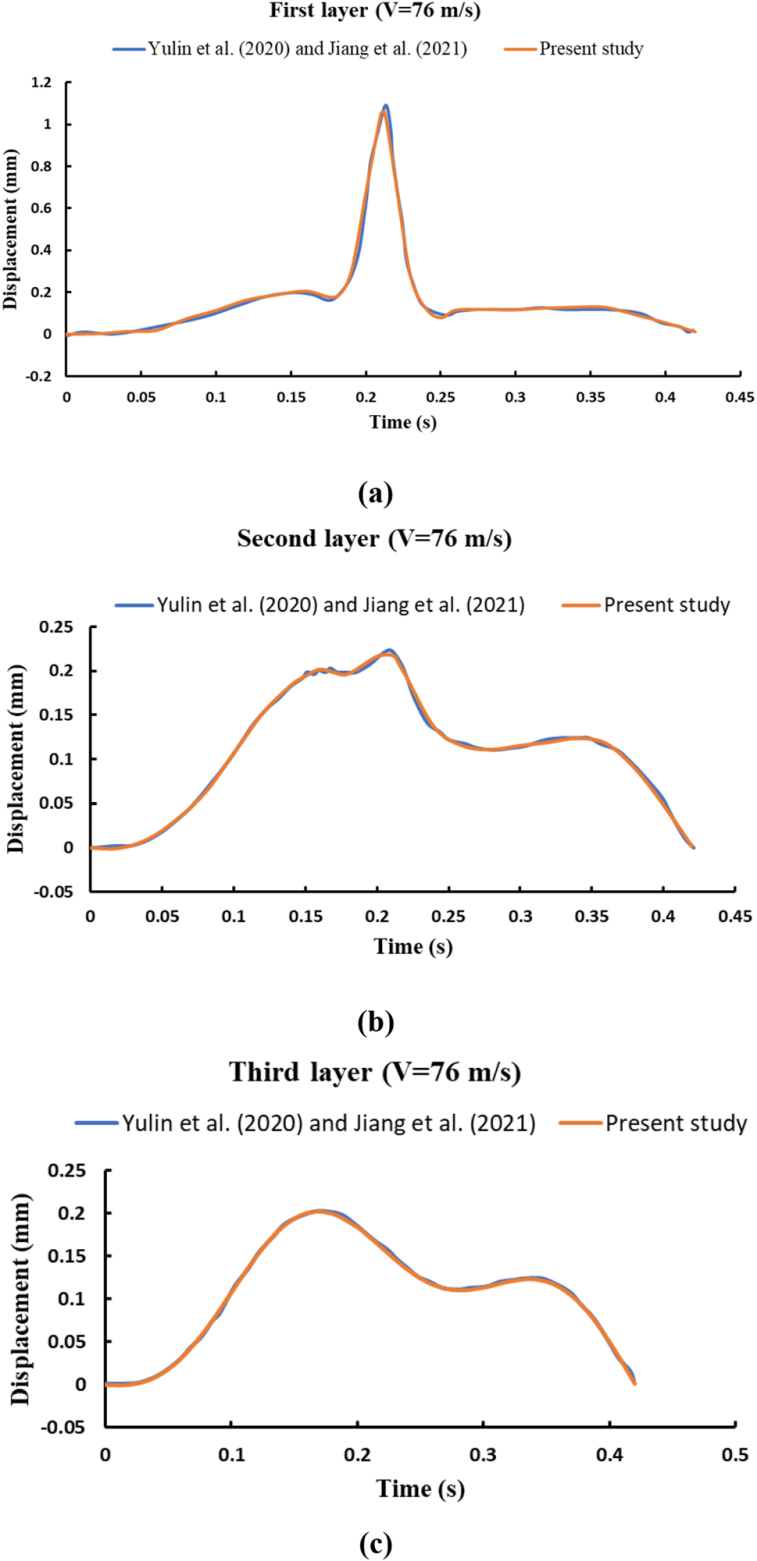
Comparison of dynamic response of triple beam system (**a**) first layer (**b**) second layer and (**c**) third layer.

The present analysis is verified with past numerical and analytical studies performed by Yulin et al.^33^ and Jiang et al.^34^. Jiang et al.^34^ investigated the dynamic response of multi-layer beams interconnected by Winkler springs subjected to moving load to simulate the railway track system. Jiang et al.^34^ verified the proposed analysis with the results of a triple beam-spring system under moving load performed by Yulin et al.^33^. Yulin et al.^33^ conducted both analytical solution and numerical analysis using ANSYS software. The detail parameters used for the analysis are listed in Table 1. The present analysis is also compared with the results of prior mentioned triple beam system. Mid-span displacement time history of all three-layer beams were recorded for moving load velocity of 76 m s^−1^ and compared with the past analytical and numerical studies as depicted in Fig. 3. Further peak mid-span displacements of three-layer beam-spring system for moving speed of 32 m s^−1^ is compared with the results obtained by Yulin et al.^33^ and is shown in Table 2. From Fig. 3 and Table 2, it is observed that all the analysis including the present one gives almost identical results which confirms the correctness of the proposed methodology.

**Table 1:** Geometric and material parameters of railway track system (Yulin et al.^33^ and Jiang et al.^34^).

Parameter	Value
*L* (m)	32
*D*_1_ (N m^2^)	66.27 × 10^5^
*D*_2_ (N m^2^)	59.50 × 10^6^
*D*_3_ (N m^2^)	35.949 × 10^10^
*m*_1_ (kg m^−1^)	60
*m*_2_ (kg m^−1^)	1275
*m*_3_ (kg m^−1^)	36,000
*K*_1_ (N m^−2^)	60 × 10^6^
*K*_2_ (N m^−2^)	90 × 10^7^
*F*_max_ (N)	85,000
*V* (m s^−1^)	76

**Table 2:** Comparison of peak mid-span displacements of three-layer beam-spring system for moving speed of 32 m s^−1^.

First layer (mm)			Second layer (mm)			Third layer (mm)		
Yulin et al.^33^		Present study	Yulin et al.^33^		Present study	Yulin et al.^33^		Present study
Analytical	Ansys results		Analytical	Ansys results		Analytical	Ansys results	
1.0638	1.0717	1.0432	0.2092	0.2091	0.1987	0.1752	0.1749	0.1752

**Figure 4:**
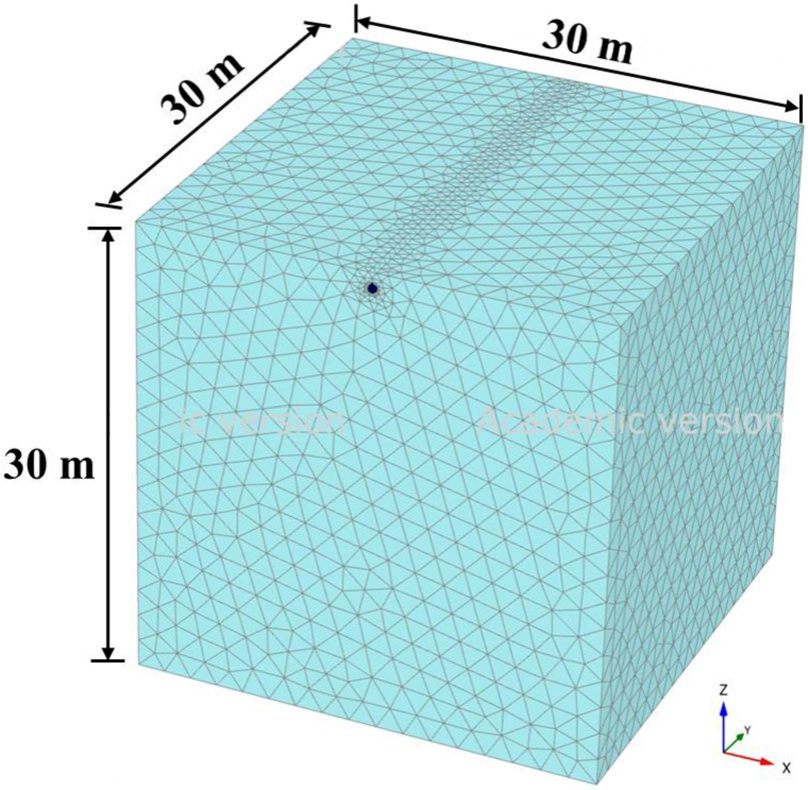
Mesh discretization of three-dimensional finite element model.

**Figure 5:**
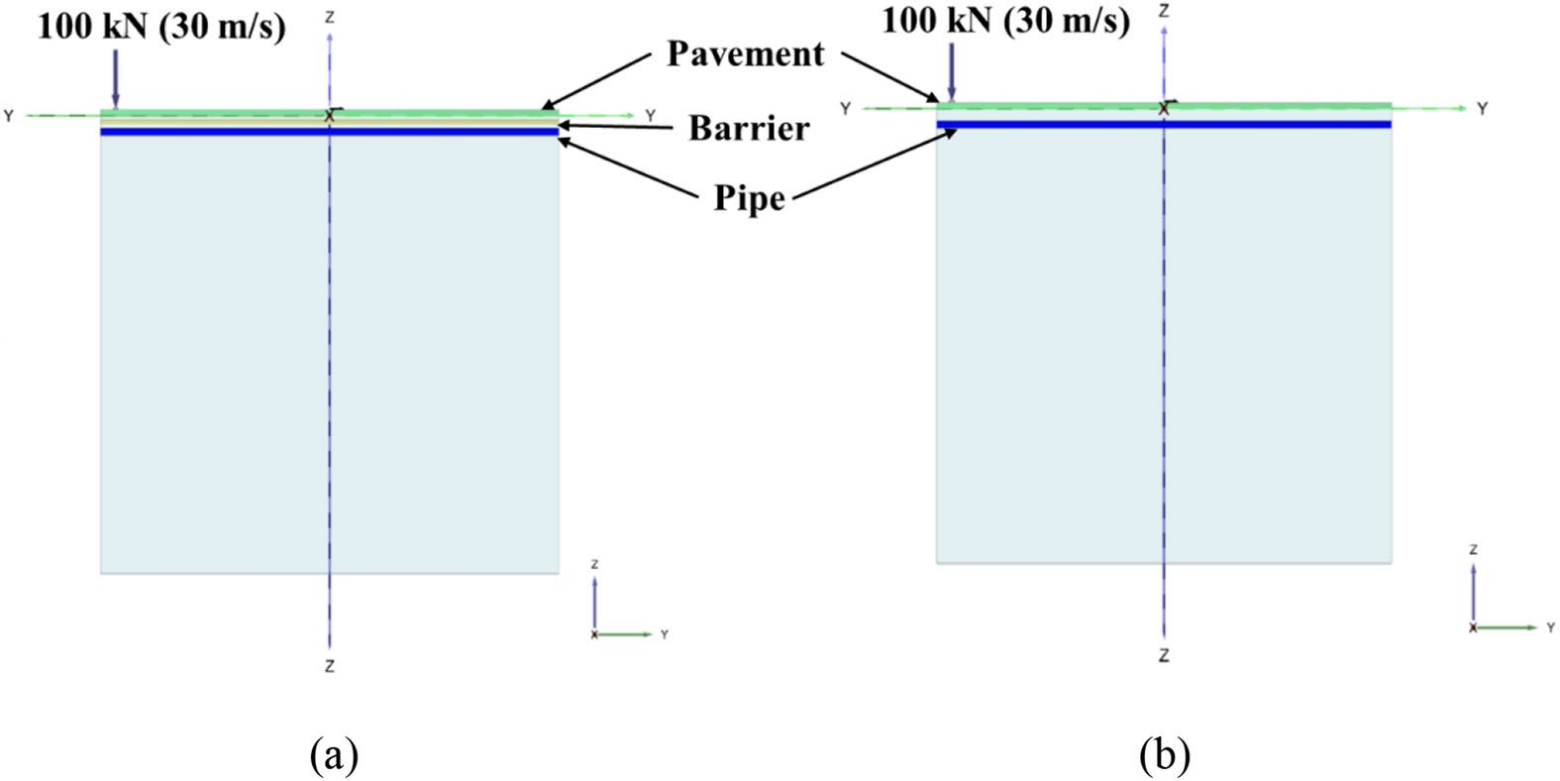
Cross-sectional view of (**a**) pipe with safeguard and (**b**) pipe without safeguard below road pavement subjected to 100 kN traffic load with moving speed of 30 m s^−1^.

Moreover, in the present study a three-dimensional finite element based numerical analysis is performed using PLAXIS 3D to validate the proposed analytical formulation. The soil, pavement layer and the barrier layer have been modeled using 10-noded tetrahedral elements. Plate elements are used to model the buried pipeline. A typical soil domain with model dimensions and mesh discretization is shown in Fig. 4. To perform the numerical analysis a traffic load of 100 kN having speed of 30 m s^−1^ is considered. Both the cases namely, the pipe with and without intermediate barrier system as depicted in Fig. 5 are simulated in the present study. The adopted material parameters for performing the numerical analysis are listed in Table 3. The side boundaries of the numerical model are restricted to move in the normal directions and the bottom boundary is fixed in all three directions. Fine mesh is used in PLAXIS model after performing mesh sensitivity study. The numerical analysis has been carried out in three phases. In the initial phase (k0 procedure) geostatic stress is defined. In second phase (plastic) pipe, barrier layer and the pavement layer are constructed. In the final stage (dynamic) traffic load has been assigned. Peak mid-span pipe displacements are recorded for both the cases. Table 4 shows the comparison of numerically obtained peak pipe responses with the results procured from the proposed analytical formulation. From Table 4 it can be observed that the analytical formulation provides overestimation in results due to the simplified assumption of beam-spring model. However, the variation between 3D numerical analysis and analytical study is acceptable which further confirms the validity of the proposed closed-form analytical study.

**Table 3:** Values of input parameters used in the validation and parametric study.

Parameter	Value
Pipe material	PVC
Pipe outside diameter (m)	0.50
Pipe wall thickness (mm)	9.50
Modulus of elasticity of pipe (GPa)	3.30
Density of pipe (kg m^−3^)	1380
Traffic load (kN)	100
Traffic velocity (m s^−1^)	30
Young’s modulus pavement (MPa)	284.40
Thickness of pavement (m)	0.40
Mass per unit length of pavement (kg m^−1^)	1250
Young’s modulus barrier, RC slab (MPa)	22,360.68
Thickness of barrier (m)	0.30
Mass per unit length of barrier (kg m^−1^)	375
Spacing between pavement and pipe for both cases (m)	0.80
Spacing between pavement and barrier (m)	0.25
Spacing between barrier and pipe (m)	0.25
Soil type	sand
Unit weight of soil (kN m^−3^)	20
Young’s modulus of soil (MPa)	30
Poisson’s ratio of soil	0.30
Damping ratio	0.05

**Table 4:** Comparison of peak mid-span pipe displacements.

Pipe with safeguard (mm)		Pipe without safeguard (mm)	
Present study (PLAXIS 3D)	Present study (Analytical)	Present study (PLAXIS 3D)	Present study (Analytical)
2.08	2.1167	3.443	4.1183

## Parametric study

The benefit of providing intermediate safeguard and the influence of traffic load and traffic velocity on buried pipe response considering both barrier and without barrier system has been investigated in the subsequent sections. The soil, pipe, barrier, pavement and traffic parameters are listed in Table 3.

### Effect of intermediate barrier layer on pipe response

**Figure 6:**
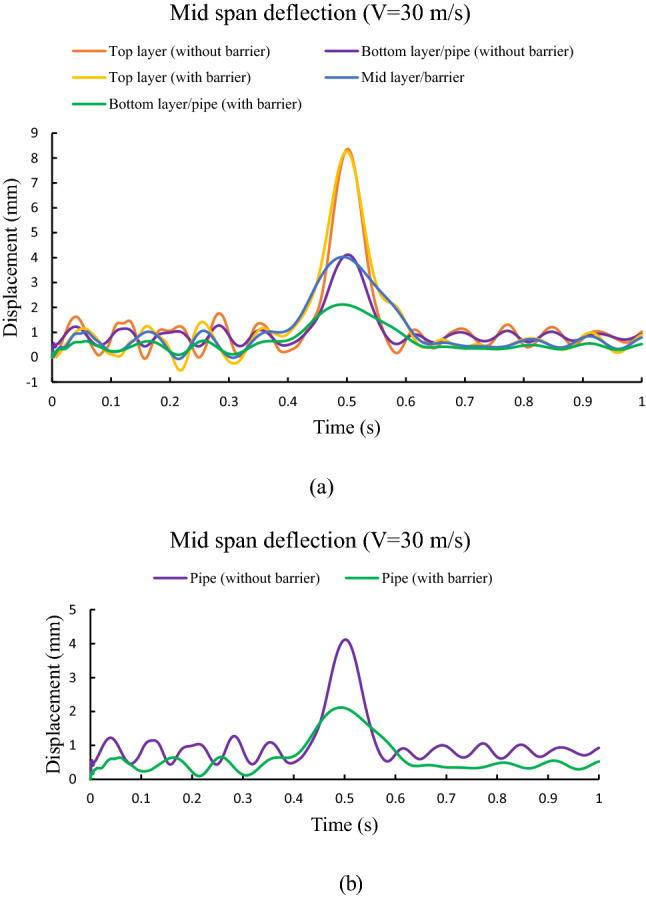
Mid span deflection time history of (**a**) all the beams and (**b**) bottom beam (pipe) for both with and without barrier system.

Mid-span deflection time history for both triple beam (with barrier) and double beam (without barrier) system subjected to moving traffic load with velocity 30 m s^−1^ are shown in Fig. 6a. It is noticed that peak deflection is observed for top beam (pavement layer) and minimum deflection is observed for the bottom beam (pipe) for both the cases. The deflection of the pipe is further reduced due to the presence of intermediate barrier system as shown in Fig. 6b. For instance, peak pipe displacement is reduced from 4.12 to 2.12 mm due to the barrier layer.

### Effect of traffic loads

**Figure 7:**
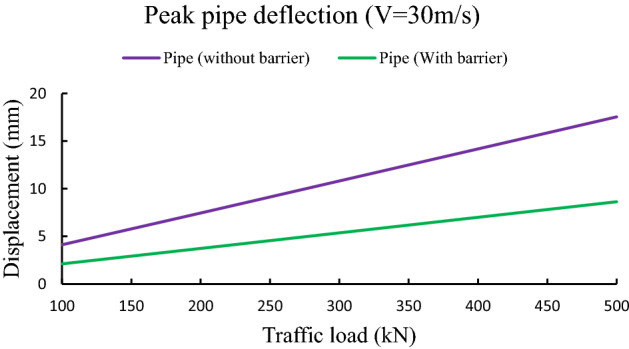
Influence of traffic load on peak pipe deflection.

To study the impact of traffic loads on pipe response, traffic load is increased from 100 to 500 kN with an increment of 50 kN keeping all other parameters are fixed as shown in Table 3. From Fig. 7, it is observed that peak pipe deflection is increases with increasing traffic loads. Further, the deflection of pipe with barrier system is less compared to pipe deflection without any protective layer. For example, pipe deflection without any barrier is increased from 4.12 to 17.54 mm for increasing traffic load from 100 to 500 kN. Further, for traffic load of 500 kN, peak pipe displacement is reduced from 17.54 mm to 8.63 mm due to the presence of intermediate barrier layer.

### Effect of traffic velocity

**Figure 8:**
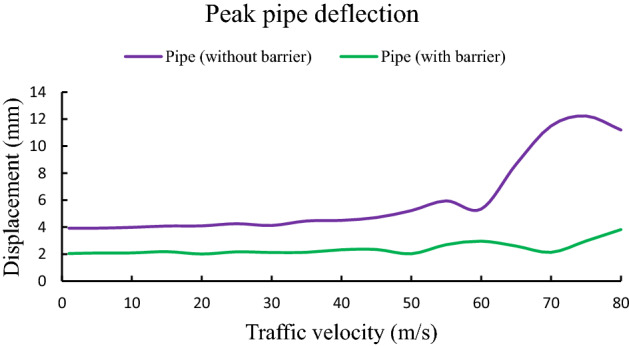
Influence of traffic speed on peak pipe deflection.

To understand the influence of traffic velocity on pipe response, velocity is increased up to 80 m s^−1^ keeping all other parameters are constant. From Fig. 8, it is noticed that the impact of velocity on peak pipe deflection is negligible up to a certain extent of traffic velocity. After that pipe displacement is increases with increasing velocity. For the case of barrier system peak pipe displacement is always less compared to pipe displacement without any barrier for all range of traffic velocity. For instance, at speed 70 m s^−1^, peak pipe displacement for with and without barrier system are 2.13 mm and 11.49 mm respectively. However, considering the feasible range of highway traffic speed, it can be concluded that the influence of traffic velocity on peak pipe response is negligible.

## Summary and conclusions

The current study proposed closed-form analytical solutions for buried pipe with and without intermediate defensive layer below road pavement. The pavement structure, intermediate safeguard layer and pipe are modelled using Euler Bernoulli’s beam. Traffic load is considered as moving point load. The soil is idealized as viscoelastic foundation with shear interaction between individual springs. The self-weight of the soil is also considered in the study. The finite sine Fourier transform, Laplace transform and their inverse are used to solve the obtained coupled governing differential equations. The proposed formulation is verified with the past numerical and analytical studies performed by Yulin et al.^33^ and Jiang et al.^34^. The analytical study is also validated with 3D finite element based program PLAXIS 3D. Further, a parametric study is conducted to investigate the benefit of using intermediate safeguard layer and the impact of traffic load and traffic velocity on buried pipe’s response. Following are the salient inferences from the present study:

- The proposed simplified formulation can be used to inspect the response of buried pipe subjected to traffic loads.
- Suitable intermediate barrier system can be provided to protect the pipelines from traffic loads.
- Pipe deformation is increases with increasing traffic loads.
- The influence of traffic speed on pipe response is negligible considering the possible range of highway traffic speed.
- At very high range of traffic velocity (> 60 m s^−1^), pipe deformation is significantly rises with increasing the traffic speed.

The study can be further refined considering the plasticity and non-linearity of pipe and soil materials. The effect of ground water table can also incorporate as future scope of work. Further, pipe can be better replicated using shell elements instead of beam as beam is not able to simulate the pipe ovalization phenomenon.

## Data Availability

The datasets generated during and/or analysed during the current study are available from the corresponding author on reasonable request (Matlab Code).
